# Metal exposure in a child population after a mine tailings dam failure. Projeto Bruminha

**DOI:** 10.1590/1980-549720230017

**Published:** 2023-02-20

**Authors:** Aline de Souza Espindola Santos, Renan Duarte dos Santos Saraiva, Ana Paula Natividade de Oliveira, Michele Alves Costa, Herling Gregorio Aguilar Alonzo, Délio Campolina, Leiliane Coelho André, Sérgio Viana Peixoto, Volney de Magalhães Câmara, Carmen Ildes Rodrigues Fróes Asmus

**Affiliations:** I Universidade Federal do Rio de Janeiro – Rio de Janeiro (RJ), Brazil.; II Universidade Estadual de Campinas – Campinas (SP), Brazil.; III Fundação Hospitalar do Estado de Minas Gerais – Belo Horizonte (MG), Brazil.; IV Universidade Federal de Minas Gerais – Belo Horizonte (MG), Brazil.; V Fundação Oswaldo Cruz, Instituto René Rachou – Belo Horizonte (MG), Brazil.

**Keywords:** Metals, Children health, Mining, Disasters, Metais, Saúde infantil, Mineração, Desastre

## Abstract

**Objective:**

This study aimed to analyze the urinary concentrations of As, Cd, Pb, Hg, and Mn in children living in areas directly affected by the tailings of the Brumadinho disaster.

**Methods:**

We performed a cross-sectional descriptive study on a population of 217 children aged 0 to 6 years, living in Córrego do Feijão (CF), Parque da Cachoeira (PC), Aranha (AR), and Tejuco (TJ), enrolled in the Longitudinal Study of Child Health in Brumadinho (Minas Gerais) — Projeto Bruminha. Socioeconomic data and urine samples were collected to determine the concentration of selected metals.

**Results:**

Children living in locations not directly affected by the disaster (AR and TJ) had higher concentrations of As and Mn than those in directly affected areas (CF and PC). Additionally, children living in locations not potentially exposed to dust from tailings mud or mining activity (AR) showed higher urinary As concentrations than those potentially exposed (CF, PC, and TJ).

**Conclusion:**

Our results suggest the need to investigate possible sources of As exposure in children living in areas not directly affected by the disaster and not potentially exposed to dust.

## RESUMO

**Objetivo:**

Este estudo teve como objetivo analisar as concentrações urinárias de As, Cd, Pb, Hg e Mn em crianças residentes em localidades diretamente atingidas pelos rejeitos do desastre em Brumadinho.

**Métodos:**

Foi realizado um estudo descritivo transversal de uma população de 217 crianças de 0 a 6 anos, residentes no Córrego do Feijão (CF), Parque da Cachoeira (PC), Aranha (AR), e Tejuco (TJ) e inscritas no Estudo Longitudinal da Saúde Infantil em Brumadinho (MG) — Projeto Bruminha. Dados socioeconômicos e amostras de urina foram coletados para determinar a concentração dos metais selecionados.

**Resultados:**

Crianças residentes em localidades não diretamente atingidas pelo desastre (AR e TJ) apresentaram maiores concentrações de As e Mn do que aquelas em localidades diretamente atingidas (CF e PC). Adicionalmente, crianças residentes em localidades não potencialmente expostas à poeira da lama de rejeito ou da atividade de mineração (AR) apresentaram maiores concentrações urinárias de As do que aquelas potencialmente expostas (CF, PC e TJ).

**Conclusão:**

Nossos resultados sugerem a necessidade de se investigar possíveis fontes de exposição ao As em crianças residentes em localidades não diretamente atingidas pelo desastre e não potencialmente expostas à poeira.

## INTRODUCTION

The exponential demand for minerals has led to a rise in mining activities worldwide, especially in Brazil, one of the five leading ore exporters, whose production increased by up to 550% between 2001 and 2011^1^. However, despite its relevance to the economy, mining is closely associated with negative impacts on the environment and human health due to the generation of residues and for representing a potential danger of disasters caused by tailings dam failure^2,3^. Mining tailings usually have metal residues that can be transported to distant areas by particulate matter and river surface runoff^4–6^.

Metal pollution poses a significant threat to human health. As, Pb, Cd, and Hg are non-essential metals among the top ten toxic substances of public health concern, according to the World Health Organization^7^. These metals easily accumulate in plants and organisms in the food chain, both important sources of human exposure^8,9^. Furthermore, contaminated water and dust around communities are potential sources of human exposure through oral, skin, and inhalation routes^10^. Other metals considered essential, such as manganese, may entail risks to human health depending on dose, valence state, and individual susceptibility.

Children are more exposed to metals because they drink more water and breathe more air in relation to their body weight compared to adults^11^. Moreover, common behaviors, such as playing near the ground and hand-to-mouth contact, contribute to greater exposure^12^. Childhood is also regarded as a critical period for the development of organ systems, and metal exposure has been associated with adverse effects on the central nervous and immune systems^13,14^.

Brumadinho is one of the most important mining municipalities in the state of Minas Gerais^15^. Projeto Bruminha is a cohort study that investigates the metal exposure profile and its effects on the health of children living in areas directly affected or not by the Córrego do Feijão mine dam failure, on January 25, 2019, in Brumadinho, which caused hundreds of deaths, destroying communities and compromising the supply and feasibility of river ecosystems. This study analyzed urinary concentrations of the arsenic metalloid and cadmium, lead, mercury, and manganese metals, as well as possible sources of exposure in the study population of Projeto Bruminha.

## METHODS

### Study area

The study area of Projeto Bruminha covers rural areas in Córrego do Feijão (CF), Parque da Cachoeira (PC), Tejuco (TJ), and Aranha (AR). CF and PC were directly affected by the disaster, with houses at a distance of up to 1.5 km from the tailings mud. In these areas, residents might have been in contact with dust from mud residues since the disaster. Tejuco was included because it is downstream of an active mining area and considered potentially exposed to the dust produced by this activity; it is approximately 3.0–4.0 km from the site affected by the tailings mud. Aranha is approximately 10 km from the tailings mud and is considered an area where the population was not directly affected by the disaster and was not potentially exposed to dust from mining residues or remediation activities (Supplementary Figure 1).

### Study design and population

This is a cross-sectional study with baseline data from the Longitudinal Study of Child Health in Brumadinho (Minas Gerais) — Projeto Bruminha. Started in July 2021, Projeto Bruminha is a prospective cohort study with annual follow-up over four years. The detailed methodology of Projeto Bruminha, including sociodemographic, behavioral, and environmental characteristics of the study population, is described in the Project Protocol. All children living in CF, PC, TJ, and AR aged 0 to 6 years were considered eligible to participate in the project, producing a list of 348 children (CF: n=51; PC: n=58; TJ: n=76; and AR: n=163) provided by the Brumadinho municipal health department, and all guardians of potential participants were invited to participate in the project. From August 15 to 30, 2021, the research team recruited and evaluated 217 (62%) children, 30 (59%) living in CF, 58 (69%) in PC, 76 (64%) in TJ, and 98 (60%) in AR. Of the total number of children recruited, 197 (90.7%) had urine samples collected (CF [25/30]: 83.3%; PC [35/40]: 87.5%; TJ [47/49]: 95.9%; AR [90/98]: 91.8%). The Research Ethics Committee of Hospital Clementino Fraga Filho, Universidade Federal do Rio de Janeiro, approved this research under opinion no. 3,897,305 on December 6, 2019. Parents gave their written informed consent to their children's participation.

### Characteristics of the study population

Two trained interviewers from the research team administered a previously tested baseline questionnaire to collect information about mothers or guardians and the children regarding sociodemographic, environmental, household, and surrounding characteristics, habits, behaviors, and diet.

### Biological sample collection

A certified nurse collected urine samples at each location in health units or outbuildings. Professionals of the health units instructed the children's guardians not to use ointments in the genital area the day before collection. Urine was collected in isolated samples with at least two hours of urinary retention using a universal container (white top) in a dust-free location. A collection bag (pediatric urine collector) was used for infants. This bag was exchanged every 30 minutes up to three times. At each exchange, the genital area was cleaned with water and sterile gauze. After urine collection, refrigerated samples were sent to a laboratory with experience in measuring metal levels.

### Analytical methods

The analytical technique employed in the laboratory for measuring metal levels was inductively coupled plasma mass spectrometry (ICP-MS), using Agilent equipment, model ICPMS7850, with a method developed and validated by the AFIP technical team according to RDC302/2005 and RDC27/2012. Samples were prepared by acid dilution and the addition of internal standards. The method sensitivity for all metals (Hg, Cd, Mn, Pb, and As) was 0.1 μg l^−1^. Regarding arsenic, total arsenic was measured. The standards used were the multi-element calibration standard 2A — Agilent (8500-6940 and 8500-6940 HG) — and internal standard Mix — Agilent (5183-4681). The limit of detection (LoD) and limit of quantification (LoQ) for As, Cd, Pb, Hg, and Mn were 0.1 μg l^−1^.

Creatinine levels were measured in biochemical analyzers — Siemens Atellica® CH Analyzer — by reaction of picric acid with creatinine in an alkaline medium (Jaffé Procedure). The analytical sensitivity of the test is determined by the value 3.0 mg dl^−1^, which corresponds to the LoQ for urine.

### Quality analysis

The analytical batches for metal measurement had their analytical performance monitored by five internal quality control levels — three controls prepared by the laboratory at pre-established concentration levels and two commercial controls by the ClinCheck or PNCQ brand. The maximum allowable variation for each control is 15%, according to RDC27/2012. During the analysis of project samples (Jul/2021 to Feb/2022), the mean monthly coefficient of variation (CV%) of As, Cd, Hg, Pb, and Mn were 5.3, 5.7, 7.1, 6.0, and 5.6%, respectively. Urine creatinine levels were monitored by two commercial control levels of the Biorad brand, with a maximum allowable CV% of 5.5%. Throughout the analysis, creatinine CV% was 2.2%.

### Statistical analysis

Urinary concentrations of As, Cd, and Hg were adjusted for creatinine values, but 25 children had urinary creatinine concentrations outside the recommended range (very diluted <0.3 g l^−1^ or very concentrated >3.0 g l^−1^ urinary creatinine) and were excluded^16,17^. The normality of data distribution was evaluated directly by histogram plots and Kolmogorov-Smirnov tests. Urinary metal concentrations did not have a normal distribution and were expressed as geometric mean (GM) with a 95% confidence interval (95%CI). Other descriptive statistics of metal concentrations included maximum, minimum, and 25^th^, 50^th^, 75^th^, and 95^th^ percentile. GM metal levels were compared according to characteristics of the study population, such as age (0–11 months, 1–2 years, >2–4 years, and >4 years), sex (male and female), skin color (white, non-white), years of maternal schooling (0, 1–9, >9 years). We also compared GM metal levels based on children's behaviors (hand-to-mouth contact, handwashing, eating dirt, eating wall paint, finger sucking, and playing with mud), socioeconomic characteristics (sewage, type of water consumed), and diet (fish consumption). Urinary metal concentrations were compared between groups using one-way ANOVA or Kruskal Wallis test for numerical variables. We considered a p-value≤0.05 statistically significant. Additionally, urinary metal concentrations were stratified by areas considered directly affected by the disaster (CF and PC) or not (TJ and AR), as well as by locations potentially exposed to mine tailings dust (CF, PC, and TJ) or not (AR). We used the Mann-Whitney test to determine potential differences between geometric metal concentrations according to the sites classified above.

## RESULTS

As, Pb, Mn, Hg, and Cd were quantified in 100, 89, 84, 64, and 23% of the samples, respectively (Table 1). Geometric urinary concentrations of As, Cd, Hg, Pb, and Mn were, respectively, 8.46 μg g^−1^, 0.11 μg g^−1^, 0.37 μg g^−1^, 0.74 μg l^−1^, and 0.40 μg l^−1^. The largest GM ranges were in As (minimum=0.70; maximum=144.30) and Mn (minimum=0.10; maximum=106.80) concentrations. The participants’ urinary concentrations of As, Cd, Hg, Pb, and Mn showed no significant differences in the four study sites (Supplementary Table 1).

**Table 1:** Metal concentrations in the study population (n=172). Projeto Bruminha, 2021.

	% >LoD	GM (95%CI)	Min-Max	Percentiles			
				25	50	75	95
As μg g^−1^	172 (100.0)	8.46 (1.08–66.41)	0.70–144.30	5.45	9.35	13.98	26.74
Cd μg g^−1^	40 (23.3)	0.11 (0.01–0.85)	0.10–0.40	0.10	0.10	0.10	0.29
Hg μg g^−1^	110 (64.0)	0.37 (0.05–2.87)	0.10–9.20	0.18	0.30	0.83	2.29
Pb μg l^−1^	153 (88.9)	0.74 (0.09–5.83)	0.10–6.00	0.40	0.80	1.30	3.63
Mn μg l^−1^	144 (83.7)	0.40 (0.05–3.17)	0.10–106.80	0.20	0.35	0.68	3.48

LoD: limit of detection; GM: geometric mean; 95%CI: 95% confidence interval; Min: minimum; Max: maximum.

Geometric Cd concentrations were significantly higher among children of younger age groups (0 to 2 years) — p=0.05 —, while Pb ones were higher in those whose guardians reported the child's skin color as non-white — p=0.04 — (Table 2).

**Table 2:** Metal concentrations stratified by socioeconomic characteristics of study participants (n=172). Projeto Bruminha, 2021.

		Cd μg g^−1^			As μg g^−1^			Hg μg g^−1^			Pb μg l^−1^			Mn μg l^−1^		
		n	GM (95%CI)	p*	n	GM (95%CI)	p*	n	GM (95%CI)	p*	n	GM (95%CI)	p*	n	GM (95%CI)	p*
Age (years)																
	0–11 months	6	0.13 (0.02–1.06)	0.05	12	10.08 (1.28–79.10)	0.19	10	0.42 (0.05–3.29)	0.44	11	0.77 (0.10–6.07)	0.37	11	0.86 (0.11–6.77)	0.07
	1–2	7	0.12 (0.02–1.11)		17	8.99 (1.15–70.53)		11	0.24 (0.03–1.85)		16	0.58 (0.07–4.58)		14	0.39 (0.05–3.06)	
	>2–4	14	0.10 (0.01–0.78)		60	7.22 (0.92–56.67)		38	0.38 (0.05–2.98)		52	0.67 (0.09–5.28)		48	0.47 (0.06–3.70)	
	>4	13	0.10 (0.01–0.78)		83	9.14 (1.17–71.73)		51	0.38 (0.05–2.99)		74	0.83 (0.11–6.55)		71	0.33 (0.04–2.55)	
Sex																
	Female	16	0.11 (0.01–0.86)	0.81	91	9.48 (1.21–74.4)	0.34	53	0.36 (0.05–2.84)	0.70	78	0.73 (0.09–5.71)	0.98	72	0.43 (0.05–3.38)	0.50
	Male	24	0.11 (0.01–0.84)		81	7.65 (0.98–60.03)		57	0.37 (0.05–2.90)		75	0.76 (0.10–5.95)		72	0.38 (0.05–2.97)	
Skin color																
	White	15	0.10 (0.01–0.78)	0.16	60	9.08 (1.16–71.25)	0.89	35	0.37 (0.05–2.94)	0.99	56	0.66 (0.08–5.16)	0.04	52	0.39 (0.05–3.07)	0.99
	Non-white	24	0.11 (0.02–0.96)		98	8.74 (1.11–68.58)		66	0.38 (0.05–3.00)		86	0.86 (0.11–6.71)		81	0.42 (0.05–3.29)	
Years of maternal schooling																
	0	3	0.11 (0.01–0.89)	0.25	6	9.17 (1.17–71.91)	0.51	4	0.41 (0.05–3.19)	0.69	6	0.76 (0.10–5.96)	0.78	6	0.42 (0.06–3.78)	0.37
	1 to 9	11	0.11 (0.01–0.84)		47	8.50 (1.08–66.73)		26	0.36 (0.05–2.83)		43	0.78 (0.10–6.13)		35	0.36 (0.05–2.82)	
	>9	25	0.10 (0.01–0.78)		114	7.51 (0.96–58.89)		78	0.32 (0.03–2.12)		100	0.60 (0.07–4.03)		99	0.29 (0.05–3.23)	

* Kruskal-Wallis or Mann-Whitney test; GM: geometric mean; 95%CI: 95% confidence interval.

GM urinary metal levels according to behavioral, environmental, and fish consumption characteristics are shown in Supplementary Table 2. The highest mean urinary Cd concentrations were detected in children whose guardians reported that they did not have the habit of washing their hands (p=0.02). In addition, As concentrations were higher in children who exhibited hand-to-mouth behavior (p=0.02), who did not eat wall paint (p=0.04), and who drank water from sources other than mineral water (p=0.01).

Children living in locations not directly affected by the disaster (AR and TJ) had higher geometric urinary concentrations of As (p=0.009) and Mn (p=0.029) than those in directly affected areas (CF and PC). Children living in AR, who would not be potentially exposed to tailings dust, had higher urinary As concentrations (p=0.009) compared to those in potentially exposed areas (Table 3).

**Table 3:** Urinary concentrations of selected metals in areas potentially affected or not by the disaster and by the dust. Projeto Bruminha, 2021.

	n (%); GM (95%CI)		p*
	Disaster^a^ (n=54)	Non-disaster^b^ (n=118)	
As μg g^−1^	54 (100.0); 6.36 (0.81–49.93)	118 (100.0); 9.64 (1.23–75.67)	0.009
Cd μg g^−1^	9 (16.7); 0.10 (0.01–0.78)	31 (26.3); 0.11 (0.01–0.87)	0.338
Hg μg g^−1^	39 (72.2); 0.36 (0.05–2.83)	71 (60.2); 0.37 (0.05–2.89)	0.807
Pb μg l^−1^	44 (81.5); 0.65 (0.08–5.10)	109 (92.4); 0.78 (0.10–6.15)	0.270
Mn μg l^−1^	46 (85.1); 0.29 (0.20–12.01)	98 (83.1); 0.47 (0.06–3.69)	0.029
	Dust^c^ (n=94)	Non-dust^d^ (n=78)	p*
As μg g^−1^	94 (100.0); 7.28 (0.93–57.11)	78 (100.0); 10.15 (1.29–79.67)	0.009
Cd μg g^−1^	21 (22.3); 0.11 (0.01–0.83)	19 (24.4); 0.11 (0.01–0.88)	0.495
Hg μg g^−1^	65 (69.1); 0.36 (0.05–2.85)	45 (57.7); 0.37 (0.05–2.9)	0.714
Pb μg l^−1^	80 (85.1); 0.80 (0.10–6.31)	73 (93.6); 0.68 (0.09–5.35)	0.204
Mn μg l^−1^	81 (86.2); 0.38 (0.05–2.99)	63 (80.8); 0.43 (0.06–3.41)	0.535

a (Córrego do Feijão — CF and Parque da Cachoeira — PC)

b (Aranha — AR and Tejuco — TJ)

c (CF, PC, and TJ)

d (AR)

* Mann-Whitney test; GM: geometric mean; 95%CI: 95% confidence interval.

## DISCUSSION

Three of the five metals analyzed in the urine of children aged 0 to 6 years — As, Pb, and Mn — were detected in more than 80% of the samples. Overall, participants from three study sites (PC, TJ, and AR) were more vulnerable, either by the report of open-air sewage disposal, dirt road housing, or water consumed from sources other than mineral, which may pose important risks to child development if associated with unfavorable environmental conditions and an inadequate dietary pattern^18,19^. Thus, in the study population, some sociodemographic conditions were associated with higher concentrations of some metals, such as higher urinary Pb levels in children whose skin color was reported as non-white by their parents. Behaviors such as hand-to-mouth contact, playing with mud and eating wall paint favor exposure to contaminated substances in the environment^20^. In our study, urinary As concentrations were higher in children who exhibited hand-to-mouth behavior, did not eat wall paint and drank water from sources other than mineral.

In general, geometric mean urinary levels of As, Pb, and Mn quantified in children living in areas directly affected by the disaster (CF and PC) were higher than in those living in a rural area in Spain (As=2.44 μg g^−1^; Pb<0.8 μg/dl; Mn<0.12 μgl^−1^)^21^. However, they were lower than urinary concentrations in child populations living in areas near copper furnaces (As=44 μg g^−1^) and mining in Mexico (As=44.5 μg g^−1^), urban areas in Malaysia (Pb=4.7 μg/dl), and an area close to a coal mine in Brazil (Pb=4.8 μg dg^−1^; Mn=0.8 μgl^−1^)^22–25^.

Sites not directly affected (AR and TJ) by the disaster showed concentrations of As and Mn significantly higher than the other locations (CF and PC). Water consumption has been considered an important source of arsenic exposure due to its physicochemical characteristics, such as solubility^26^. Our results suggest higher mean urinary As concentrations in children whose guardians declared drinking water from sources other than mineral water. Additionally, urinary As concentrations were also higher in children living in areas not directly affected by the disaster (AR and TJ) — places with the lowest mineral water consumption (data not shown). Fish consumption is also one of the main As sources^27^. In this study, fish consumption was low and did not differ between areas directly affected by the disaster or not. Therefore, we do not believe fish consumption is an important source of As exposure. Mn is found in many types of rocks and in soils from which iron ore is extracted in several cities from Minas Gerais^28^. It is also often found in groundwater, drinking water, and low-level soil, in addition to being naturally present in most foods, such as grains, vegetables, fruits, meat, fish, and nuts^29^. In addition, Mn was quantified in our study population because, according to the Ministry of Health Technical Opinion no. 5/2019-DSASTE/SVS/MS, the concentrations of this metal in all tailings mud samples analyzed were above the mean concentrations found in Brumadinho soils. In our results, the concentrations of this metal were higher in areas not directly affected by the disaster (AR and TJ). Thus, the inhalation route may not be as significant for this metal as water, although environmental studies should be carried out to investigate the exposure sources more thoroughly.

The As metalloid has been found in fugitive dust in mining areas, often associated with large particles that do not usually remain suspended for long. However, finer particles in mine tailings^30,31^ have higher arsenic content^32^. In our study, As concentrations were higher in AR, a location not potentially exposed to tailings dust. In this scenario, the inhalation route might have less impact on exposure than the oral one, of which water consumption could be a more important source.

Except for the weak but significant Pb-Mn relationship, most evaluated metals did not have a significant correlation (data not shown). Our study hypothesis was that the tailings mud from the Brumadinho disaster could be a source of metal exposure. Additionally, an active mining area would also be a source of exposure due to mechanical processes such as rock crushing. Therefore, the inhalation route would greatly contribute to metal exposure, considering that, in sites directly affected by the disaster, the population drinks bottled mineral water, and these communities do not have a livelihood activity based on fishing — which would imply oral exposure. Yet, the results of this study do not support our hypothesis, and the lack of a significant correlation for the analyzed metals may suggest, in a purely speculative way, that it needs to be better investigated and that different sources of exposure may be associated with our findings.

This study has limitations that are worth mentioning. First, the environmental concentrations of the evaluated metals were not measured. Second, no information was collected on the consumption of chicken and other foods^33^ or passive smoking in the study population, which is an important source of exposure to arsenic and cadmium. Third, the total urinary arsenic concentration does not provide information on the form of the arsenic absorbed. Fish is a significant source of organic As, while contaminated drinking water, soil, or dust are important sources of inorganic As exposure. Urinary As is the biomarker that best represents inorganic As compared to blood; however, speciation of the studied matrix could not be performed. Lastly, collecting a single urine sample instead of repeated samples over time may limit the interpretation of accurate exposure, especially for Pb and Mn, which were not corrected for creatinine levels since we cannot differentiate intra- and inter-individual variability sources of analytical factors. Despite the limitations, our findings provide information on the potential exposure of children from Brumadinho areas to toxic metals according to different exposure scenarios; namely, locations directly affected by mining tailings from the disaster caused by Córrego do Feijão B1 Mine failure, an active mining area, and another site not directly affected by the disaster or mining activity. To date, we have no knowledge of studies that evaluated metal concentrations in this context. Thus, this study can provide exposure parameters, as in Brazil, especially Minas Gerais, approximately 80% of the municipalities have mining as an economic activity.
